# The Natural History Model of Hepatitis C Virus Infection and the Economic Evaluation of Alpha Interferon Treatment

**DOI:** 10.2188/jea.12.22

**Published:** 2007-11-30

**Authors:** Kenshi Hayashida, Ichiro Nagasue, Takashi Fukuda, Atsuaki Gunji

**Affiliations:** 1 Department of Healthcare Economics and Quality Management, Graduate School of Medicine, Kyoto University.; 2 Pfizer Pharmaceuticals, Inc.; 3 Department of Pharmacoeconomics, Graduate School of Pharmaceutical Sciences, University of Tokyo.; 4 General Research Institute, Seigakuin University.

**Keywords:** economic evaluation, hepatitis C, interferon therapy, markov chain model

## Abstract

Interferon (IFN) therapy is used for the treatment of hepatitis C virus (HCV) disease, but is so expensive that it creates controversy as to whether or not it is effective use of limited health care resources. In order to make this judgement possible, it must be necessary to build a comprehensive disease model of HCV infection from social perspective. A Markov chain model of the natural history of HCV infection in male patients was developed. Parameters on the clinical phase of the disease were adopted from published reports, but those of the non-clinical phase were estimated from the data on blood donation and mortality rates from the disease. Then, adding in the modeling of treatment outcome from IFN therapy and cost-benefit analysis, IFN therapy was economically evaluated.Using this model, it was shown that (1) IFN therapy for chronic hepatitis C (CHC) would be economically beneficial at least in the Japanese situation, (2) the complete response rate to therapy would be the most sensitive factor affecting outcome, and (3) the younger the person cured by IFN therapy, the greater the benefit seen.These results demonstrate that IFN therapy would be beneficial in the case of the CHC patients (male).

## INTRODUCTION

Interferon (IFN) is now used in many countries for the treatment of Hepatitis C virus (HCV) disease but it is very expensive and it is still uncertain whether the treatment is cost/beneficial to society. Some studies^1^^-^^6^^)^ have tried to answer this question, however, they would still have uncertainties in the natural history of HCV infection, long-term treatment-response rates, and estimates of future management costs of endstage liver disease as Brown^7^^)^ and Koff^8^^)^ pointed out.

Although it is especially important to estimate disease-progression rates, it is to treat many uncertainties because 1) clinical reports are inherently limited to see disease-progression rates only after chronic hepatitis C (CHC) is diagnosed and 2) long-term prognosis cannot be tested in a conventional epidemiological manner. Thus we need a reliable and validated model of natural history of the disease in order to judge whether the IFN treatment is cost/beneficial to society. This study, therefore, aims to:

(1) develop a natural history model of HCV disease using a Markov chain,
(2) estimate the parameters, and
(3) examine the benefit of IFN treatment of male patients.

## METHODS

### Modeling

#### 1. Basic modeling

The natural history of HCV disease is modeled using a Markov Chain. The states are to be defined as a vector _i_S at time i:

$${}_{\text{i}}S={(}_{\text{i}}S_{1}{\;}_{\text{i}}S_{2}{\;}_{\text{i}}S_{3}{\;}_{\text{i}}S_{4}{\;}_{\text{i}}S_{5}{\;}_{\text{i}}S_{6})$$ (1)

where s_1_ is uninfected, s_2_ infected, s_3_ chronic hepatitis C (CHC), s_4_ liver cirrhosis (LC), s_5_ hepato-cellar carcinoma (HCC), and s_6_ dead.

The number of people at Time i in each health state in a hypothetical cohort is defined as:

$${}_{\text{i}}L={(}_{\text{i}}l_{1}{\;}_{\text{i}}l_{2}{\;}_{\text{i}}l_{3}{\;}_{\text{i}}l_{4}{\;}_{\text{i}}l_{5}{\;}_{\text{i}}l_{6})$$ (2)

**Figure 1.**
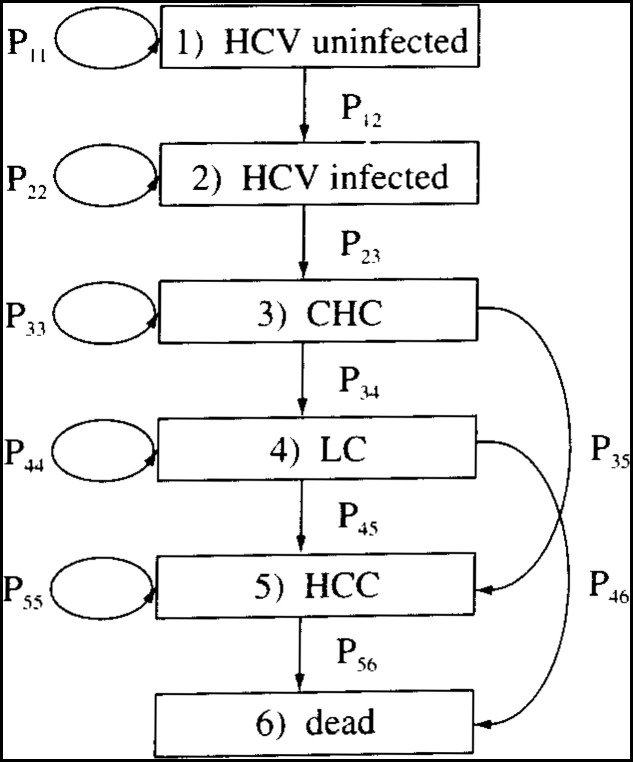
Markov chain model of the natural history of HCV disease.

The transfer probabilities of disease from Time i to Time i + 1 are described as:

$$iP=\left(\begin{array}{cccccc} P_{11} & P_{12} & 0 & 0 & 0 & 0\\ 0 & P_{22} & P_{23} & 0 & 0 & 0\\ 0 & 0 & P_{33} & P_{34} & P_{35} & 0\\ 0 & 0 & 0 & P_{44} & P_{45} & P_{46}\\ 0 & 0 & 0 & 0 & P_{55} & P_{56}\\ 0 & 0 & 0 & 0 & 0 & 1 \end{array}\right)$$ (3)

where for instance, death from the states of Infected and Chronic Hepatitis, and the disease progressions reversibility are assumed to be implausible (See Figure 1).

Therefore,

$${}_{\text{i+1}}L={}_{\text{i}}L\;{}_{\text{i}}P$$ (4)

#### 2. Death rate from causes other than HCV liver disease

The death rate from causes other than HCV liver disease between Time i to Time i + 1 can be defined as:

$$iQ=\left(\begin{array}{cccccc} {}_{\text{i}}q & 0 & 0 & 0 & 0 & -{}_{\text{i}}q\\ 0 & {}_{\text{i}}q & 0 & 0 & 0 & -{}_{\text{i}}q\\ 0 & 0 & {}_{\text{i}}q & 0 & 0 & -{}_{\text{i}}q\\ 0 & 0 & 0 & {}_{\text{i}}q & 0 & -{}_{\text{i}}q\\ 0 & 0 & 0 & 0 & {}_{\text{i}}q & -{}_{\text{i}}q\\ 0 & 0 & 0 & 0 & 0 & 0 \end{array}\right)$$ (5)

_i_q is the death rate at age i, and these data are available from the life table.

Thus,

$${}_{\text{i+1}}L={}_{\text{i}}L(E-{}_{\text{i}}Q){}_{\text{i}}P$$ (6)

Or

$${}_{\text{i+1}}L={}_{\text{i}}L\;{}_{\text{i}}P(E-{}_{\text{i}}Q)$$ (7)

where E is the unit matrix. Equation (6) assumes that death from all other causes occurs soon after the _i_L is observed at Time i and then the state of the population transfers with the probability _i_P. Equation (7) means the state transfers first and the death occurs all at once before the observation at time i+1. The reality will be somewhere in-between, but for the purpose of simplicity, calculation (6) is adopted in this report.

#### 3. Modeling of treatment outcome from IFN therapy

It has been accepted that a positive response to IFN can be classified into two possibilities^9^^)^ : complete response and partial response. Complete response is defined as both histologically efficacious (i.e., persistently normal serum ALT level for at least six months after normalization occurring no later than six months after the end of treatment) and virologically efficacious (i.e., a negative serum HCV RNA at least for six or more months after completion of therapy). Partial response is defined as either histologically or virologically efficacious.

The complete responders are assumed to be cured, and the partial ones are assumed to have transferred back from CHC to the carrier state. Accordingly, chronic hepatitis C patients, if treated with IFN, may 1) become cured by the rate r_31_, 2) have persistent viremia (r_32_), or 3) remain in the same state (1+r_33_). Modeling of the treatment effect is possible by introducing a matrix R as follows:

$$R=\left(\begin{array}{cccccc} 0 & 0 & 0 & 0 & 0 & 0\\ 0 & 0 & 0 & 0 & 0 & 0\\ r_{31} & r_{32} & r_{33} & 0 & 0 & 0\\ 0 & 0 & 0 & 0 & 0 & 0\\ 0 & 0 & 0 & 0 & 0 & 0\\ 0 & 0 & 0 & 0 & 0 & 0 \end{array}\right)$$ (8)

where *r*_31_ + *r*_32_ + *r*_33_ = 0

Therefore, the population of the cohort with IFN therapy becomes:

$${}_{\text{i}}L^{\prime }={}_{\text{i}}L(E+R)$$ (9)

#### 4. Modeling of direct costs (medical costs)

Medical costs depend on the place of care, clinical states, and the type of treatment they receive. This model assumes that all the patients who are in the states, CHC, LC and HCC are under some form of treatment. Thus, these patients are either inpatients or outpatients, whose treatment costs vary as do their different clinical states. Furthermore, each patient may change his or her status of physician visits from outpatient to inpatient, or vice versa, but the overall ratio of inpatients and outpatients can be fixed.

Given that IFN therapy cost (C_IFN_) and conventional treatment costs (C) are;

$$C_{\text{IFN}}=(0\;0\;c_{\text{IFN}}\;0\;0\;0)$$ (10)

$$C_{\text{IFN}}=(0\;0\;c_{3}\;c_{4}\;c_{5}\;0),$$ (11)

treatment costs for all the patients at age i with IFN and conventional ones can be described as the inner product of two vectors;

$${}_{\text{i}}{LC}_{\text{IFN}}={}_{i}l_{3}c_{\text{IFN}},$$ (12)

and

$${}_{\text{i}}LC={}_{i}l_{3}c_{3}+{}_{i}l_{4}c_{4}+{}_{i}l_{5}c_{5}.$$ (13)

#### 5. Modeling of indirect costs

Persons _i_L can create a value of _i_B per person year. Wage depends on age, geographical area, industry type, size of organization, educational background, and so on. But in this study, for the purpose of simplicity, indirect cost is equated to the average wage lost per person at each age. The total value, therefore, that the persons at age i create annually can be described as

$${}_{\text{i}}{LW}_{\text{i}}B$$ (14)

where W is a diagonal matrix (w_j_), each element of which is the employment rate in each state j.

### Estimation of parameters

#### 1. Disease progression (for 5 years)

It is of pivotal importance for the validity of the model to estimate the parameters as precisely as possible, especially in estimating the elements of the transfer probability matrix in this model. The parameters of the clinical stages are available from the published literature based on clinical observation and follow-up, but those of non-clinical stages, i.e. p_12_ and p_23_ do not exist. They were estimated, therefore, by methods described later in detail.

##### (1) P_56_ (HCC→dead)

This means the probability of death of the HCC patient in 5 years. The survival rate in HCC is periodically surveyed and reported^10^^)^ by the Japanese Liver Cancer Study Group for each type of treatment. Based on this nationwide survey, the five year survival rates were 40.8% (n=9099) for cases of liver ablation, 8% (n=7633) for cases of transcatheter arterial embolization (TAE) and 0% for cases of other treatments from 1978 to 1991. In a recent survey on patients treated between 1990 and 1991 (n=11916), the frequency of treatment type was found to be as follows: liver ablation - 33.4%; TAE - 35.5%; others - 31.1%. The overall five-year survival rate, therefore, was adjusted to take account of these recent treatment patterns, using the weighted average of each group.

The five-year survival rate with HCC was calculated to be 16.5% and thus P_56_ was estimated to be 0.835, on condition that available data includes not only HCV-related HCC, but also all others, and that this factor is not taken into account for this analysis.

##### (2) P_45_ (LC→HCC) and P_46_ (LC→dead)

Based on 795 LC patients (followed-up from 2 to 17 years), Ikeda^11^^)^ reported that the accumulated risk of progression to HCC in 5 years is 19.4%.

Kato^12^^)^ followed up 401 LC patients (mean 4.4 years) and found the risk in 5 years to be 20.8%.

Tsukuma^13^^)^ followed up 240 LC patients for four years and concluded the accumulated risk of progression in three years was 12.5%. This was adjusted to 20% for progression risk in 5 years, assuming that LC progressed to HCC in a constant rate for these years.

The results calculated from the three reports cited above were quite similar and so the average weighted by the number of cases was taken for the estimate. P_45_ was estimated to be 0.199. Ikeda^11^^)^ reported P_45_ as 0.215 from 349 cases, all of which were diagnosed as HCV antibody positive. This means the value is very close to the estimated P_45_.

Ikeda^11^^)^ reported the survival rate of LC in the same cohort was 84.1% for five years after the first diagnosis. The five-year mortality with LC was, therefore, estimated to be 0.159 (P_46_).

The disease progression of these stages is reported to vary by gender, smoking and drinking habits^13^^)^, which were neglected in this estimation.

##### (3) P_34_ (CHC→LC) and P_35_ (CHC→HCC)

Takase^14^^)^ reported a retrospective study on 169 CHC patients whose progression of disease were observed during the past 23 years. The literature suggests that the probability of progressing from CHC to LC within a ten-year period would be 26%. Assuming that the disease progresses constantly, the progression rate in five years P_34_ was estimated to be 0.14.

Takase^14^^)^ concluded that 11.8% of CHC patients would develop liver cancer in a ten-year period. Assuming that the probability of progressing from CHC to HCC is constant over the period, P_35_ was estimated to be 0.061 in five years.

Tsukuma^13^^)^ also followed 677 CHC patients for four years and found that a three-year total risk of developing HCC from CHC was described as 0.038 of the cohort. Assuming that it is constant over the period of the illness, this finding^13^^)^ can be converted to a five year progression rate of 0.0625. These two figures were almost identical and a weighted average of the two was taken. The result was 0.0624 for P_35_.

Takase^14^^)^ stratified the probability by histological activity determined by liver biopsy, but the histological differences were not taken into consideration for this estimation.

As in estimating P_45_ and P_56_, gender and habits of smoking and drinking were neglected for the purpose of simplification although they could have been factored.

##### (4) Estimation of P_12_ (Uninfected→Infected) and P_23_ (Infected→CHC)

Two processes were taken for this purpose. The first step mentioned here was to estimate roughly the level of the values for further exact inference of P_12_ and P_23_. For that purpose, supposing that blood donors would be either 1) Uninfected or 2) Infected, data^15^^)^ from the Japanese Red Cross on the HCV positive rate of blood donors were used. Since the data needs to represent the cohort precisely, the data measured before the Notification of HCV infection (June, 1992) was used. The used Red Cross data was based on C100-3 antibody, and the actual HCV carrier rate (i.e., the rate of HCV RNA positive) was adjusted by a ratio of 82.6%, based on the literature^16^^)^ that addressed correlation between C100-3 antibody assay and direct qualitative analysis of HCV RNA.

It is assumed that the probability of infection with HCV (P_12_) is constant regardless of age, and that it had brought about the gradual increase of positivity rate with age.

The two parameters P_12_ and P_23_ were estimated by minimizing the sum of squares of the differentials between simulated figures and the actual cross-sectional data of blood donors. For the purpose of this calculation, a matrix which is a part of P was used.

$$\left(\begin{array}{ccc} P_{11} & P_{12} & 0\\ 0 & P_{22} & P_{23}\\ 0 & 0 & 1 \end{array}\right)$$ (15)

where male data between age 16 and 19 represents a default value.

Due to the small size of the donor population, those in age sixties looked irrelevant for the estimation of transition probability. Also, the actual data showed that the population over age 50 had much higher rate of the infection than those under age 50. Therefore, estimating the parameters was based on the donor population under fifties.

**Figure 2.**
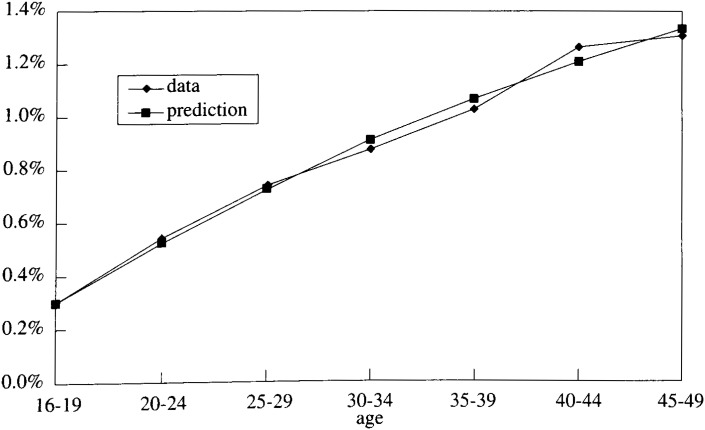
The rate of HCV RNA positive.

Calculations were performed with the Macro-program of Excel. P_12_ and P_23_ were estimated to be 0.0026 and 0.11 respectively (See Figure 2).

##### (5) Model fitting

**Figure 3.**
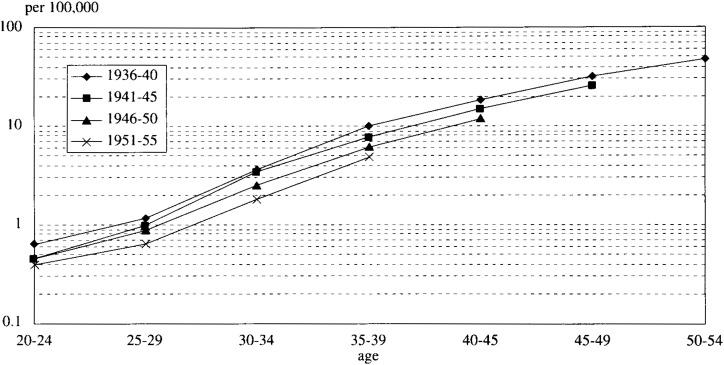
The mortality rate of LC and HCC (4 cohorts born in each period).

Using all the parameters estimated by the methods mentioned above, it was determined whether or not the model could successfully explain the cohort mortality rates by age which were taken from the vital statistics. For the use in comparison, the adjustment of the two census data was as follows:

The mortality of HCC was based on the study^10^^)^ that looked into the percentage of HCC out of liver cancer and that of HCV infection in HCC patients. The mortality of LC was similarly based on the related literature^11^^)^. In fact, four male cohorts were extracted: those born between 1936-40, 1941-45, 1946-50 and 1951-55. The mortality rates of these cohorts are plotted in Figure 3. The shapes seemed quite similar but the younger the cohort, the lower the levels.

**Figure 4.**
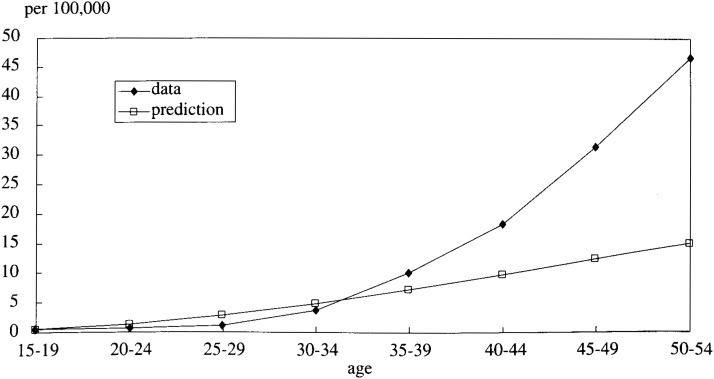
The mortality rate of LC and HCC (compared to a cohort born in 1936-40).

The mortality rates simulated by the model were compared with that of the cohort born between 1936-40 and they were plotted in Figure 4. As shown in this figure, the difference between simulated and real data curves widened as age increased.

It was assumed that this mismatch resulted from the hypothesis that P_23_ is a constant which is independent of time. It was postulated, therefore, that P_23_ is a logistic function and actually a logistic function as follows:

$$P_{23}=1/(1+\exp(at+b))$$ (16)

P_12_ was postulated to be constant over ages but different between cohorts.

Starting from the initial value mentioned above, estimation of the parameters, a, b and P_12_ were executed by the least squares method that is essentially curve fitting of the simulated data to that of cohort born between 1936-40. The Macro-program ‘Solver’ (Excel) was used again for this purpose. Next, fixing a and b, only P_12_ was changed to fit the simulated data to other cohorts.

In Figure 5-1, 5-2, 5-3, 5-4

**Figure 5-1.**
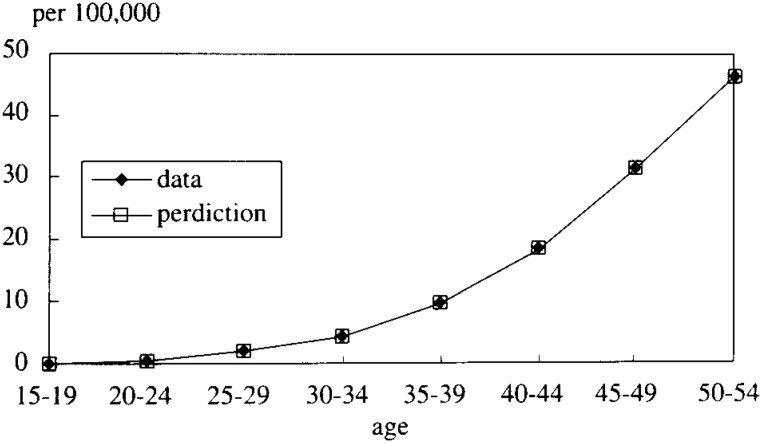
The mortality rate of LC and HCC (cohort born in 1936-40).

**Figure 5-2.**
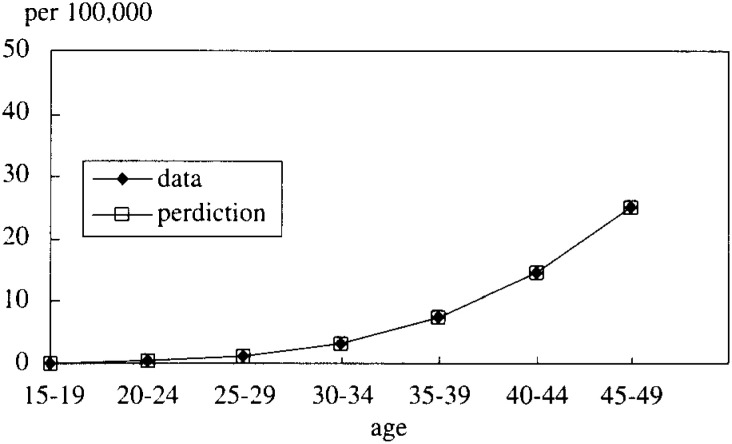
The mortality rate of LC and HCC (cohort born in 1941-45).

**Figure 5-3.**
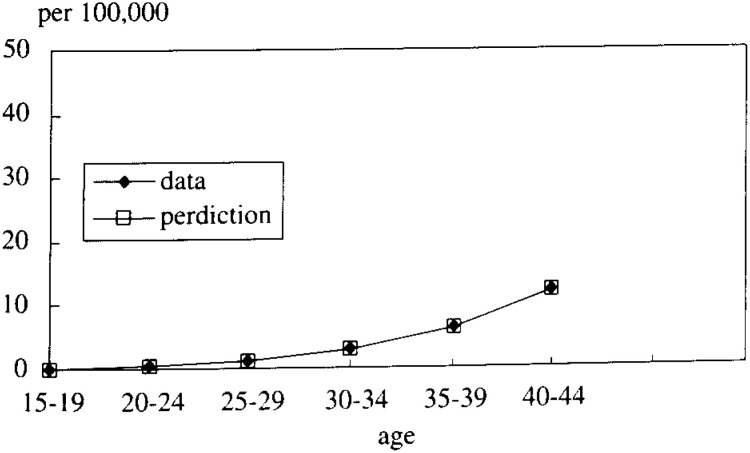
The mortality rate of LC and HCC (cohort born in 1946-50).

**Figure 5-4.**
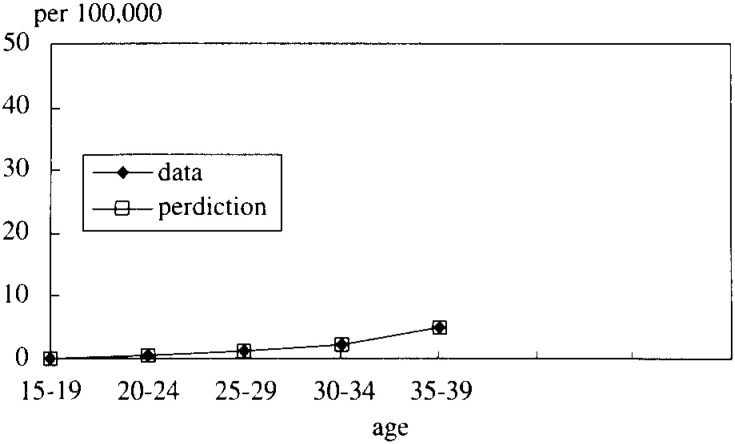
The mortality rate of LC and HCC (cohort born in 1951-55).

, the simulated data fitted very well with the real mortality data. The estimated results were;

$$P_{23}=1/(1+\exp(-0.1273t+4.6725))$$

$$\begin{array}{rl} P_{12} & =\text{0.0036 for the cohort born between 1936-40}\\ & =\text{0.0029 (1941-45)}\\ & =\text{0.0023 (1946-50)}\\ & =\text{0.0018 (1951-55)} \end{array}$$

These results strongly support the correctness of the hypotheses.

##### (6) Interpretation about the data from the Japanese Red Cross by reestimated P_12_ and P_23_

There was a estrangement between the data from the Japanese Red Cross on the HCV positive rate of blood donors^15^^)^ and the HCV positive rate estimated from the four cohorts, on condition that blood donors would be either 1) Uninfected or 2) Infected .

**Figure 6.**
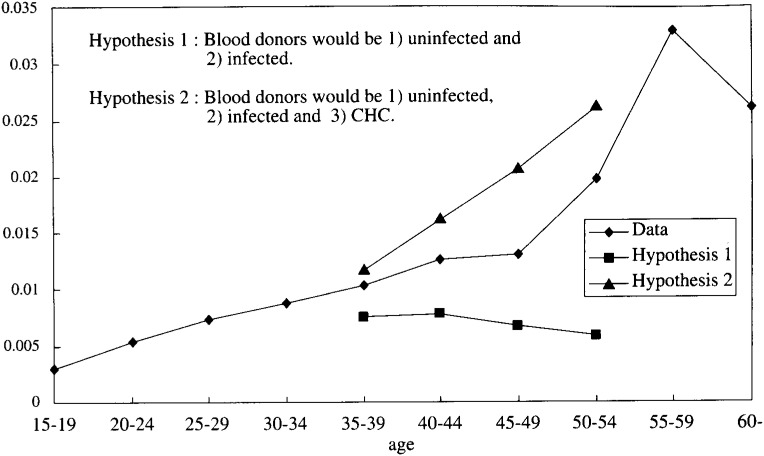
The rate of HCV RNA positive.

However, it has been reported^17^^)^ that slightly less than a half of all CHC patients are not symptomatic. It could be considered that blood donors would be included CHC patients. Thus, by including the hypothesis, i.e. blood donors would be 1) Uninfected or 2) Infected or 3)CHC, real data exist between these two hypotheses and there is no contradiction on the interpretation (See Figure 6).

##### (7) Estimation of parameters for females

It is thought to be impossible that the parameter of disease progression is assumed to be the same between male and female. This is because both HCV positive rates of blood donors are similar while the mortality rates of LC and HCC are different.

Thus, P_12_ and P_23_ for female were reestimated by using the mortality rates of LC and HCC in the same way as for male. It was assumed that the female probability of infection with HCV(P_12_) is constant regardless of age and to be the same as male.

The result was, as Figure 7-1, 7-2, 7-3, 7-4

**Figure 7-1.**
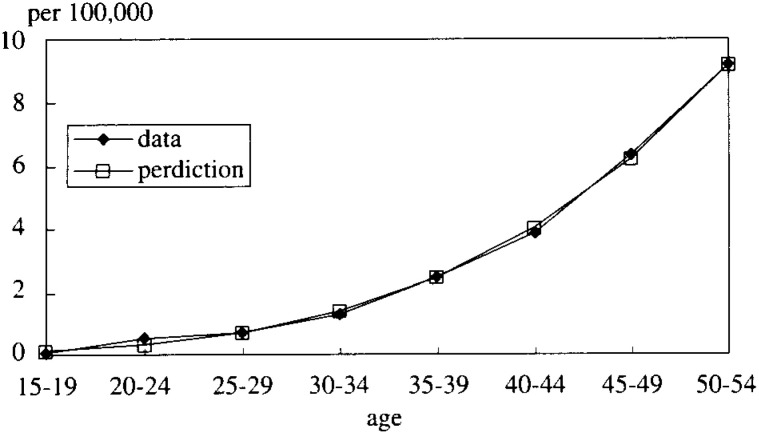
The mortality rate of LC and HCC (cohort born in 1936-40).

**Figure 7-2.**
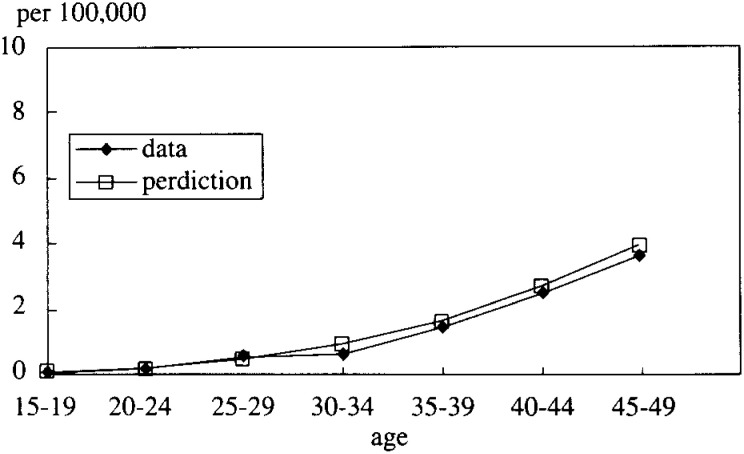
The mortality rate of LC and HCC (cohort born in 1941-45).

**Figure 7-3.**
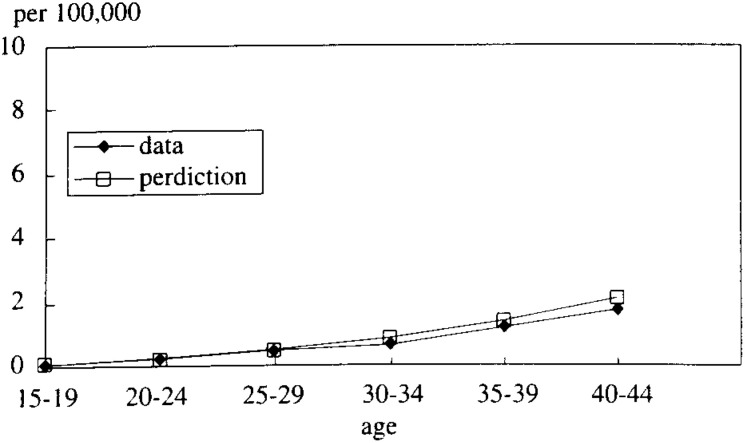
The mortality rate of LC and HCC (cohort born in 1946-50).

**Figure 7-4.**
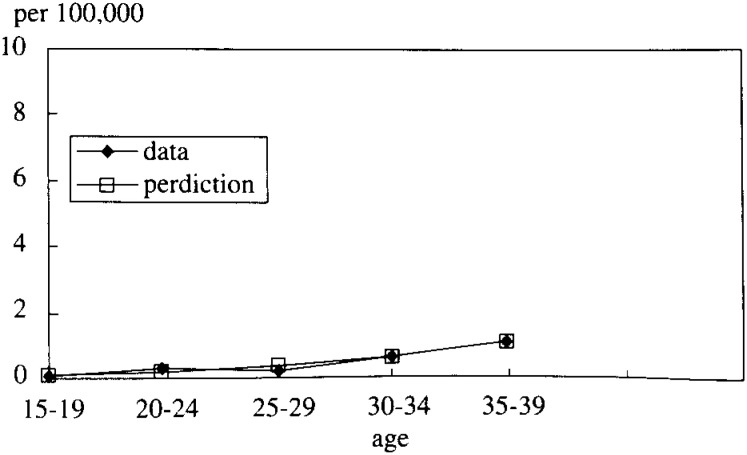
The mortality rate of LC and HCC (cohort born in 1951-55).

shows, that female P_23_ was estimated to be different logistic function from that of male. And, the simulated data fitted well with the real mortality data. P_23_ = 1/(1+exp(-0.0456t + 4.739))

#### 2. Calculation of treatment outcome from IFN therapy

Efficacy of IFN was based on the phase III clinical trials^9^^)^ of recombinant IFN alpha-2b in CHC. In this trial, CHC patients were received IFN treatment of 600 MIU (10 MIU 6 times a week for 4 weeks and 3 times a week for 12 weeks). Complete response rate was 35.7% and partial response rate was 11.9%.

#### 3. Calculation of costs and benefits

As described in the modeling of direct costs, medical costs are dependent on the place of care, clinical states and the type of treatment they receive. Conventional costs for CHC and LC were obtained from the Social Medical Expense Survey^18^^)^. Inpatient costs for HCC were based on the bills for HCC patients from six facilities. Outpatient costs were not accessible but considered similar to those for LC since a health related QOL survey(S. Fukuhara, personal communication) complying with the SF-36 guideline indicated that these two conventional costs were not significantly distinct. The cost of IFN treatment was calculated based on its National Health Insurance price as of April 1994.

The ratio of inpatients and outpatients were estimated by using the Patient Survey^19^^)^. The number of male outpatients were derived from the following formula.

The whole sum of outpatients

= the number of new outpatients + that of outpatients who reconsulted and who did not do so on the investigation day

= the number of new outpatients + that of outpatients who reconsulted on the investigation day × an average consultation interval × 6/7 (6/7 is referred in literature^20^^-^^23^^)^).

Ratios of inpatient and outpatient were calculated in the following manner:

The total number of the outpatients with CHC would be:

1100+40600×6.7×6/7=234260

while that of the inpatients was counted as 5900. Therefore, the ratio of inpatient and outpatient in CHC became 2.46 to 97.54. In an identical way, the ratio in LC was calculated as 9.94 to 90.06 since:

400+12100×7.3×6/7=76111

and the inpatient number was 8400. As for HCC patients, the data was adapted from the QOL survey (S. Fukuhara, personal communication) complying with the SF-36 guideline (See Table 1).

**Table 1.** Medical cost per day.

	Outpatient		Inpatient	
CHC	$4.28	(97.54%)	$46.92	(2.46%)
LC	$5.51	(90.06%)	$74.88	(9.94%)
HCC	$5.51	(44.44%)	$154.35	(55.56%)

IFN	$15.80/1MIU			

The numbers inside parenthesis are ratios of inpatients and outpatients.

Data source: Prescription survey of Japan (Ministry of Health and Welfare); Patient survey of Japan (Ministry of Health and Welfare)

Using those figures, the total treatment costs borne by the cohort at Time i:

= the number of CHC patients at Time i × (0.9754 × 4.28 × 365 + 0.0246 × 46.92 × 365)

+ that of LC patients × (0.9006 × 5.51 × 365+0.0994 × 74.88 × 365)

+ that of HCC patients × (0.4444 × 5.51 × 365 + 0.5556 × 154.35 × 365)

This model includes the patients with various state from the Uninfected to the HCC having different levels of commitments to their job. Here in this model, all the 1) Uninfected and 2) Infected people were assumed to be a full-time worker. Patients with other states of the diseases (i.e., CHC, LC, and HCC) were estimated to be based on the QOL survey(S. Fukuhara, personal communication). The employment rate of LC inpatients was absent and was presumed to be similar to that of LC outpatients (See Table 2). Male wages were calculated based on the ‘95 Wage Statistics^24^^)^ published by the Ministry of Labor. The average annual wages by age group are shown in Table 3. Therefore, the total labor values at time i should be equal to that of the total employed patients at time i multiplied by the average annual wages.

**Table 2.** Employment rate .

	Outpatient	Inpatient
CHC	88%	83%
LC	73%	73%
HCC	75%	50%

Data are taken from QOL survey.

**Table 3.** Male average wage.

Age	Wage/month
18-19	$ 912
20-24	$1,086
25-29	$1,331
30-34	$1,607
35-39	$1,846
40-44	$2,053
45-49	$2,234
50-54	$2,270
55-59	$2,023

Data source : Wage statistics of Japan (Ministry of Labor)

## RESULTS

### Cost-benefit analysis

With the assumptions and the models discussed above, the IFN treatment was economically evaluated by cost-benefit analysis.

First, a base case of 100,000 of 0 year old cohort without HCV was assumed and was followed every five years. P_12_ was set at 0.0018, which is the value of the cohort born between 1951-55. Secondly, estimated population for each age group was adopted as observations for analysis. Then, the following two sets of cohorts were compared to the balance between costs and benefits: cohorts (A) without and (B) with interventions by IFN therapy. The intervention was supposed to be done only once at the age 20, 30,40, or 50. The first set of the cohort (A) progressed with probability P, while the second set (B) was intervened by IFN only once before progressing with probability P.

Medical costs and wages were accumulated until each age group becomes 60 years old. As for medical costs, while (A) owed conventional costs only, (B) incrementally paid the cost of IFN. As for wages, some limitations in using the Wage Statistics included a lack of data available for the people age between 16 and 17 and on the definitions of age groups. Both limitations were hence reasonably adjusted.

Using these prices, the direct, indirect and total benefit produced per person of the cohort were calculated at the present value with a discount rate of 5% annually.

Table 4 shows the results of the calculations of the direct, indirect and total benefit produced per person of the cohort who received IFN treatment at age 20, 30, 40 or 50. These results show that IFN treatment is economically beneficial to society and that it is more beneficial when the treatment is given at an earlier age. Also, although P_12_ was set at the different value, the results of the benefit analyses were quite identical.

**Table 4.** Benefit of each strategy.

Age	Direct benefit	Indirect benefit	Total benefit
20	18,612	49,747	68,359
30	14,818	39,072	53,891
40	8,440	22,582	31,021
50	-2,136	5,884	3,748

The unit is $/patient.

### Sensitivity Analysis

Each parameter estimated above was varied over a one percent change of value to determine the impact on total benefit. In other word, the percent change of total benefit was calculated by each parameter multiplied by 1.01.

Only the results which exceeded a 0.5% change to 1% increase for each parameter are shown in Table 5. The most sensitive parameters common to all age groups were found to be the efficacy of IFN and employment rate of the CHC outpatient. For patients treated at the age of 50 years, outcome was more sensitive to changes of the parameters.

**Table 5.** Sensitivities of each parameter on the outcome.

Parameter	Age: 20	30	40	50
Medical cost for the HCC inpatient				0.95%
IFN cost				-2.41%
P_35_(CHC→HCC)				1.11%
Complete Response rate	0.93%	0.99%	1.12%	2.93%
Employment rate of the CHC outpatient	-0.75%	-1.05%	-1.60%	-5.93%
Employment rate of the LC outpatient				-1.09%
Male average wage for 55-59 years				1.57%

Only the results which exceeded a 0.5% to 1% increase for each parameter are listed.

Overall, the sensitivity analysis did not negate the conclusion that IFN therapy would be beneficial in cases of CHC patients (male).

## DISCUSSION

### 1. Natural history of HCV infection

In this study, there remains some room for argument in estimating parameters.

For instance, all parameters were estimated under the hypothesis that P_23_ would be a logistic function and that P_12_ would be constant over ages but different between cohorts, and that others would be constant being independent of time. The hypothesis, however justified, could very well explain the end result of the disease, i.e. the mortality rates by age of cohorts. It is also an important fact that this fitting has become possible by introducing a hypothesis that the putative probability P_23_ is a function of time and the shape is a logistic function, and that only the levels of P_12_ are different among generations. Thus the natural history model of HCV infection in this study, which is different from previous studies^1^^-^^6^^)^, would be an original disease model verified by the reliable data, i.e. the mortality rates of LC and HCC and the HCV positive rate of blood donors.

The reliable data of the infection rates of HCV is becoming available through the recent reports^6^^,^^25^^)^. If the values in fact go beyond the range supposed by the sensitivity analysis of this report, then re-evaluation must be done but the program developed by this study will do it very easily.

Statistical methods for meta-analysis were not used and most of the values were estimated simply by taking weighted averages using the number of patients followed-up. In the case of P_34_ and P_35_, as the value for exact five years was not available, they were estimated by assuming that the probability remains constant over the period and converting the exponent into five years.

Furthermore, as it is well known that the disease may be influenced by drinking and smoking habits^13^^)^, it is quite natural that the course of the disease may well vary between individuals. Also, the data used in estimating parameters were not resulted from only HCV related disease. Thus, it would be necessary in future to take such factors into account in this analysis.

### 2. Economic evaluation and sensitivity analysis

The result of benefit analysis is influenced by many factors. Particularly, the efficacy of IFN treatment and the employment rates of patients affects those results.

Since IFN *α*-2b is used on a world-wide basis, the results described here may be applicable elsewhere other than Japan. It is well known that this is dependent on the HCV genotype^26^^)^, the level of virus in the blood^9^^)^ and on the doses and duration of treatment^27^^)^.

The employment rates of patients and the wage levels will be quite different in different locations. Although the result of the benefit analysis is quite difficult to generalize, it is important that the different strategies for treatment can be examined by this approach, to determine how much each benefits the society.

The benefit also heavily depends on the indirect cost. In this study, however, total benefit was mainly discussed. Thus, when only direct benefit is taken up, as table 4 shows, that of the treatment of those treated in their 20’s, 30’s and 40’s turns out to be plus. It means that IFN therapy for CHC in 20’s, 30’s and 40’s is economically beneficial.

The end-point of calculations was set at 60 years old, which is obviously arbitrary. To drag the end-point beyond the age, however, would inevitably require further assumptions, for example, on how to estimate the welfare expense and relevance of the discount rate etc.

It was assumed that all CHC and LC patients were under treatment. It has been reported^17^^)^ that slightly less than a half of all CHC patients are not symptomatic. It is likely, therefore, that those CHC patients who are unaware of their disease would not seek medical attention; further, there are those amongst the CHC patient population who are not motivated to visit a physician. As the evaluation is based on the difference between conventional treatment and IFN, however, this factor is canceled out.

### 3. Application for overseas condition

This paper has firstly demonstrated that the natural history of HCV infection can be successfully modeled by Markov chain and secondly that it can be used for benefit analysis. The result of benefit analysis in the context of Japanese situation proved that the treatment of CHC (male) by IFN is beneficial to society. Further debate, however, will be necessary before generalizing this conclusion to other parts of the world.

As it is already known that HCV genotype distributions will differ among different areas, it is also plausible that the natural history of the disease will also differ between areas.

Even in other areas of the world, however, if clinical experience is well documented and vital statistics on the mortality rates by age for the disease are available, the same approach will be possible and cost-benefit analysis would become possible. In other words, the findings of this model apply only to Japan because it is based exclusively on Japanese data on benefits and costs, however the methodology could be used elsewhere.

## CONCLUSION

In this study, the natural history of HCV infection disease, the effect of interventions by IFN treatment, and the costs and benefits of treatments were modeled, and IFN treatment for CHC patients was economically evaluated by using the models at age limit of 60 years.

From the results of these analyses, it is concluded that:

(1) Generally, IFN treatment for CHC would be economically beneficial in Japan(male), (2) the complete response rate to the therapy is the most sensitive factors on the result, (3) the younger the person treated by IFN is, the higher benefit would be brought about.

## References

[1] Marcellin P, Boyer N, Gervais A, . Long-term histologic improvement and loss of detectable intrahepatic HCV RNA in patients with chronic hepatitis C and sustained response to interferon-*α* therapy. Ann Intern Med, 1997 ; 127: 875-881. 9382365 10.7326/0003-4819-127-10-199711150-00003

[2] Bennett WG, Inoue Y, Beck JR, Wong JB, Pauker SG, Davis GL. Estimates of the cost-effectiveness of a single course of interferon-*α* 2b in patients with histologically mild chronic hepatitis C. Ann Intern Med, 127: 855-865. 9382363 10.7326/0003-4819-127-10-199711150-00001

[3] Kim WR, Poterucha JJ, Hermans JE, Therneau TM, Dickson ER, Evans RW, Gross JB Jr. Cost-effectiveness of 6 and 12 months of interferon-*α* therapy for chronic hepatitis C. Ann Intern Med, 1997; 127: 866-874. 9382364 10.7326/0003-4819-127-10-199711150-00002

[4] Moriguchi H, Sato C. The socioeconomic evaluation of strategical treatment for chronic hepatitis C with interferon. Jpn J Health Econ Policy, 1996; 3: 169-178. (in Japanese)

[5] Wong JB. Cost-effectiveness of treatments for chronic hepatitis C. Am J Med, 1999; 107(6B): 74S-78S. 10.1016/s0002-9343(99)00388-5 10653463

[6] The Japan Society of Hepatology. Kangan Hakusho. 1999. (in Japanese)

[7] Brown JL. Efficacy of combined interferon and ribavirin for treatment of hepatitis C. Lancet, 1998 ; 351: 78-79. 9439486 10.1016/S0140-6736(05)78157-5

[8] Koff RS. Interferon-*α* for chronic hepatitis C: reducing the uncertainties. Ann Intern Med, 1997; 127: 918-920. 9382371 10.7326/0003-4819-127-10-199711150-00011

[9] Iino S, Hino K, Kuroki T, Suzuki H, Yamamoto S, Ogawa N. Clinical study of treatment with interferon *α*-2b in patients with chronic active hepatitis C. Kiso To Rinsho, 1996; 30: 57-93. (in Japanese)

[10] The Liver Cancer Study Group of Japan. Eleventh primary liver cancer in Japan 1990-1991. 1994. (in Japanese)

[11] Ikeda K, Saitoh S, Koida I, . A Multivariate analysis of risk factors for hepatocellular carcinogenesis: a prospective observation of 795 patients with viral and alcoholic cirrhosis. Hepatology, 1993; 18: 47-53. 7686879

[12] Kato Y, Nakata K, Omagari K, . Risk of hepatocellular carcinoma in patients with cirrhosis in Japan. Analysis of infections hepatitis viruses. Cancer, 1994; 74: 2234-2238. 7922974 10.1002/1097-0142(19941015)74:8<2234::aid-cncr2820740805>3.0.co;2-6

[13] Tsukuma H, Hiyama T, Tanaka S, . Risk factors for hepatocellular carcinoma among patients with chronic liver disease. N Engl J Med, 1993; 328: 1797-1801. 7684822 10.1056/NEJM199306243282501

[14] Takase K, Nakano T, Tameda Y, Kosaka Y. Histological findings of the liver. Nippon Rinsyo, 1995; 53 (Suppl): 662-666. (in Japanese) 7563853

[15] Yoshizawa H, Tanaka J, Ohhori K, . Prevalence of the HCV associated markers among blood donors in Japan. Nippon Rinsyo, 1991; 49: 357-365. (in Japanese) 1901371

[16] Yoshizawa H. Blood transfusion and hepatitis C. In; S Hattori, ed. Hepatitis C medical. Medical View, Tokyo, 1994: 103-107. (in Japanese)

[17] Kiyosawa K, Tanaka E, Sodeyama T, Furuta S. Chronic hepatitis C, liver cirrhosis. In; Furuta S, Suzuki H, eds. Hepatitis C update, 1993: 129-139. (in Japanese)

[18] Ministry of Health and Welfare. Prescription survey of Japan. 1993. (in Japanese)

[19] Ministry of Health and Welfare. Patient survey of Japan. 1990. (in Japanese)

[20] Nakamura Y, Hashimoto S, Koike S, . References about estimates of total number of patients based on patient survey of Ministry of Health and Welfare. Kosei No Shihyo, 1994; 41(5): 3-9. (in Japanese)

[21] Hashimoto S, Nakamura Y, Koike S, . Method of estimates of total number of patients based on patient survey of Ministry of Health and Welfare. Kosei No Shihyo, 1994; 41(6): 3-12. (in Japanese)

[22] Koike S, Imamura T, Onodera S, . Validity of total number of patients based on patient survey of Ministry of Health and Welfare. Kosei No Shihyo, 1994; 41(8): 9-15. (in Japanese)

[23] Nakamura Y, Hashimoto S, Koike S, . Study of application of total number of patients based on patient survey of Ministry of Health and Welfare. Kosei No Shihyo, 1994; 41(10): 26-33. (in Japanese)

[24] Ministry of Labor. Wage statistics of Japan. 1995. (in Japanese)

[25] Sasaki F, Tanaka J, Moriya T, . Very Low incidence rates of community-acquired hepatitis C virus infection in company employees, long-term inpatients, and blood donors in Japan. J Epidemiol, 1996; 6: 198-203. 9002386 10.2188/jea.6.198

[26] Yuki N, Hayashi N, Kasahara A, . Pretreatment viral load and response to prolonged interferon-*α* course for chronic hepatitis C. J Hepatol, 1995; 22: 457-463. 7665863 10.1016/0168-8278(95)80109-x

[27] Yokosuka O, Kato N, Hosoda K, . Efficacy of longterm interferon treatment in chronic liver disease evaluated by sensitive polymerase chain reaction assay for hepatitis C virus RNA. Gut, 1995; 37: 721-726. 8549952 10.1136/gut.37.5.721 PMC1382881

